# Social Media Use and Academic Performance in Chinese Children and Adolescents: A Moderated Chain Mediation Model

**DOI:** 10.3390/bs14100867

**Published:** 2024-09-25

**Authors:** Yu Hou, Chuting Qin, Peng Xu

**Affiliations:** 1 School of Journalism and Culture Communication, Zhongnan University of Economics and Law, Wuhan 430073, China; yhou90@zuel.edu.cn (Y.H.); qinchuting@stu.zuel.edu.cn (C.Q.); 2 Department of Sociology, School of Philosophy, Zhongnan University of Economics and Law, Wuhan 430073, China

**Keywords:** social media use, academic performance, online learning behavior, prosocial behavior, family socioeconomic status

## Abstract

Based on data from the 2022 Chinese Minors’ Digital Life and Online Protection Survey, this study investigated the status quo of social media use and its influencing mechanism on academic performance among Chinese children and adolescents. The statistical results indicate that the average level of Chinese students’ social media use was generally low, with their academic performance varying across socio-demographic and schooling characteristics. After controlling for other variables, it was found that the frequency of social media use could exert a significant positive impact on students’ academic performance. Moreover, the mechanism analysis revealed that online learning behavior and prosocial behavior served as chain mediators linking social media use to academic performance. Specifically, students could transfer their social media behavioral patterns to the internet-based learning context, and then effectively utilize remote learning resources. Meanwhile, engagement with social media would cultivate individuals’ prosocial personality, thereby stimulating intrinsic motivation for learning and ultimately enhancing academic performance. The heterogeneity analysis further confirmed that the impact of social media use on students’ academic performance was stronger in lower-class families, underscoring the moderating role of family socioeconomic status in the relationship between social media use and academic performance. The findings suggest that if academic performance is regarded as an integral part of individual capacity development, then the rational utilization of social media resources might be a pivotal approach to alleviate the predicament of developmental inequality faced by students from low socioeconomic backgrounds.

## 1. Introduction

Students’ academic performance is a net outcome primarily determined by personal cognitive and non-cognitive attributes, as well as the sociocultural environment in which the socialization process occurs [1,2]. Extensive empirical research has underscored the significance and multifaceted impacts of academic performance among school-aged children and adolescents. Specifically, some scholars pointed out that a strong academic performance not only alleviated the psychological stress caused by failure or underperformance [3] but also bolstered individuals’ self-assurance and mitigated issues, such as academic procrastination and psychological disorders [4]. Conversely, inadequate academic performance could lead to a heightened level of academic pressure experienced by adolescents, resulting in disrupted sleep patterns, suboptimal dietary habits, and a range of negative physiological responses [5,6]. Moreover, from a macroscopic perspective, academic performance also appears to be intricately intertwined with the sustainable development of the labor market. First, it could serve as a metric for evaluating problem-solving skills, which were significant predictors of the participants’ quality in the youth labor market. In fact, it was found that students with higher academic performance were more likely to receive better employment opportunities and higher salaries in subsequent career trajectories [7]. Additionally, as a crucial criterion for competitive selection within the education system, academic performance, played a pivotal role in the distribution and allocation of talents among different types of schools, which could exert an unignorable influence on the labor market structure [8]. Therefore, it is imperative to thoroughly examine the current state and influential factors of academic performance in order to promote students’ comprehensive development.

Among the various factors associated with academic performance, internet-related behavior is considered one of the most influential determinants in the digital era. In fact, reinforced by the widespread adoption of internet technology and rapid digitization, significant transformations have taken place in both the demographic composition of Chinese internet users and their patterns of online behavior over the past decade. According to China’s 53rd Statistical Report on Internet Development, 18.5% of China’s 1.092 billion internet users fell within the age range of 6 to 19 years old, with more than 95% of this group engaging in social media for instant communication and online entertainment activities [9]. Given the pervasive nature and popularity of social media among Chinese younger audiences, it is crucial for both public discourse and academia to pay close attention to its potential impacts on students’ academic development [10]. Admittedly, the accessibility of social media indeed fostered educational equity by providing children and adolescents with unrestricted access to a diverse range of free learning resources, which held particular significance for rural youth in China [11]. Furthermore, the convenience offered by social media platforms also empowered users to generate and publish digital content online, resulting in an abundant repository of user-generated resources. Through the personalized and collaborative learning platforms, Chinese students could actively participate in knowledge exchange and effectively utilize high-quality educational materials, thereby facilitating the resolution of learning challenges while enhancing academic performance [12].

In addition, it is worth noting that the potential influence of social media use on academic performance does not manifest immediately and often requires specific mediating mechanisms to exert more substantial effects. To be specific, some researchers found that there were certain correlations linking online learning behavior and prosocial behavior with students’ educational development [13,14]. However, few studies have systematically analyzed the mediating roles of online learning behavior and prosocial behavior in the relationship between social media use and academic performance, particularly in non-Western contexts, such as Chinese society. As a result, it is necessary to incorporate these two behavioral factors into the analytical framework to further elucidate micro-behavioral mechanisms underlying how social media affects Chinese students’ academic performance. Moreover, previous research has mainly focused on specific age groups (e.g., junior school students), neglecting a comprehensive analysis of social media use among minors aged 6 to 18. In fact, there might be age-related heterogeneity in students’ cognitive and behavioral development from childhood to late adolescence [15]. In this study, we intend to address the above-mentioned oversights through using the data from a nationwide survey on Chinese minors’ digital life and online protection. In particular, we quantitatively investigate the mediating roles of online learning behavior and prosocial behavior, as well as the moderating role of family socioeconomic status between social media use and academic performance. From the perspective of Bourdieu’s sociological theory, the field of study practice is not reducible to the issue of individual learning attitudes or decisions, whilst any “choice” of study behaviors ought to be affected by both individual agency and social structural factors (e.g., online media environment) [16]. In this sense, our endeavor to investigate the mechanisms linking social media use and academic performance would yield significant policy implications for the advancement of children and adolescents within the context of online and offline interactive learning environments.

## 2. Literature Review and Research Hypotheses

### 2.1. Social Media Use and Academic Performance

Social media generally serves as a network tool that facilitates individuals to share opinions, experiences, and perspectives in the digitalized society. It primarily encompasses social networking sites, microblogs, forums, and other forms of online communication. In comparison to traditional media channels, such as newspapers and television, social media exhibits remarkable advantages in terms of immediacy and interactivity. Hence, social media per se not only expands people’s access to information resources but also significantly reduces the opportunity cost associated with information transmission. Additionally, it has given rise to “virtual communication”, which enhances the potential for collaborative actions through social networks [17]. Meanwhile, children and adolescents, often referred to as “digital natives”, typically show an inherent inclination toward various forms of social media. For example, it has been found that more than 90% of American teenagers employ mobile devices for internet access while sending and receiving an average of more than 60 social messages per day [18]. As such, social media holds the potential to play a pivotal role in the educational development of children and adolescents within the digital social environment.

Empirical studies conducted across different countries have indicated an inconclusive relationship between social media use and academic performance. To be specific, the majority of the existing literature supports the positive impact of social media on enhancing academic performance, particularly through its provision of networked and mobile learning opportunities for teenagers [19]. With the convenience offered by mobile intelligent devices, students are able to engage in ubiquitous and active learning, which could create favorable conditions for their educational development [20]. Additionally, the highly participatory and interactive nature of social media also empowers students to devise personalized learning trajectories, which would be beneficial for enhancing academic performance [21]. Additionally, the judicious utilization of social media ought to enhance students’ self-efficacy in the learning process, which would help to promote a passion for learning and improve study efficiency [11,22,23]. Nevertheless, some scholars have still highlighted that excessive use of social media might encroach upon study time, thereby impeding students from adhering to their original learning schedules and exacerbating academic burdens [24,25,26,27,28]. Evers’s analysis further revealed that sleep disorders arising from the use of social media could disrupt daytime learning efficiency and contribute to burnout, which established a cyclic relationship among social media, burnout, and low academic performance [29].

Theoretically speaking, the inconsistent findings mentioned above may partly stem from variations in the sociocultural milieu that individuals inhabit. In Western societies, parents tend to adopt a more open and inclusive stance toward their children’s internet usage and study behaviors. This parenting style could potentially amplify children’s exposure to inappropriate online content through social media platforms and hinder their academic performance. In contrast to Western societies, the act of fostering children’s pursuit of education has long been esteemed as a prestigious familial endeavor in traditional Chinese culture. At present, the uncertainty surrounding economic development and the scarcity of high-quality resources have intensified Chinese parents’ awareness of educational competition. In the meantime, the existing educational institutions still adhere to a comprehensive ability-based selection mechanism, leading to evident stratification within the fundamental education system. Consequently, parents from diverse social backgrounds inevitably face the predicament of ensuring their children’s access to education [30]. In order to ensure exceptional performance in the high school and college entrance examinations, Chinese parents could endeavor to optimize social media as a valuable resource for promoting students’ academic achievements. Furthermore, the Chinese government has recently advocated for “Online Clear Action”, designed to foster a healthy online environment for children and adolescents. Thus, under the influence of Chinese family and social structure, social media use is more likely to play a positive role in students’ academic development. Therefore, considering the localized sociocultural context, the following hypothesis is proposed:

**H1:**  *Social media use has a positive impact on academic performance in Chinese children and adolescents.*

### 2.2. The Mediating Role of Online Learning Behavior

Online learning behavior refers to the learning activities conducted by learners using internet media platforms, highlighting their behavioral features of self-directedness and intrinsic motivation. In accordance with the life course theory, the primary aim of individual socialization is to facilitate minors’ secure development through intergenerational support while nurturing their autonomous personality traits during childhood and adolescence [31]. In this regard, online autonomous learning aligns with the objective of fostering autonomy through socialization. Nowadays, as digital technology becomes increasingly integrated into daily life, socialization is no longer confined to face-to-face interactions but gradually from offline to online scenarios. Thus, the tension between the dual objectives of “secure growth” and “independence” becomes increasingly salient [32]. Notably, children and adolescents can now easily construct interdependent relationships with their peers through mobile phones or other internet access devices. This situation may augment parental challenges in directly supervising their offspring. In fact, due to the inherent openness and anonymity of the internet itself, parents and teachers commonly possess limited knowledge regarding this young group’s online communication activities, so the lack of supervision undoubtedly engenders safety concerns throughout their online behavioral process [33]. Prior studies have demonstrated that the utilization of social media could enhance individuals’ digital literacy, strengthen their awareness and capacity to acquire online knowledge, and foster an exploratory learning habitus [11,12]. Moreover, social media use could also enhance individuals’ perception of academic self-efficacy, thereby fostering a more favorable psychological inclination toward learning [34]. Therefore, this study proposes the following hypothesis:

**H2a:**  *Social media use has a positive impact on online learning behavior in Chinese children and adolescents.*

Additionally, online learning behavior can also serve as a predictor of academic performance. For instance, Spitzer and Musslick carried out an empirical study harnessing a dataset of over 2500 K-12 students in Germany and revealed a positive correlation between online learning behavior and students’ academic achievement [13]. This association was partially attributed to the fact that online learning behavior facilitated access to abundant educational resources by promoting personalized learning approaches [21], which would be conducive to fostering broader connections with unfamiliar peer groups for knowledge exchange [35,36]. Based on the above analyses, we posit that social media use may indirectly influence academic performance through the mediating pathway of online learning behavior. Accordingly, the following hypotheses are put forward:

**H2b:**  *Online learning behavior has a positive impact on academic performance in Chinese children and adolescents.*

**H2c:**  *Online learning behavior plays a mediating role in the relationship between social media use and academic performance in Chinese children and adolescents.*

### 2.3. The Mediating Role of Prosocial Behavior

Prosocial behavior encompasses the range of actions exhibited by individuals during social interactions, including acts of assistance, cooperation, and sharing. This specific form of behavior generally conforms to social norms and demonstrates an individual’s conscientiousness, comprehension, and active involvement in public affairs [37]. In the digital age, an individual’s social media usage should closely pertain to real-life behavioral patterns [38,39]. For example, Boulianne conducted a meta-analysis and presented that the utilization of social media platforms had a substantial positive impact upon social engagement behaviors [40]. There are three possible explanations for this phenomenon. Firstly, from a psychological perspective, social media platforms actually offer an interactive online environment that can elicit moral identification and motivation among young users, which is beneficial for the development of offline prosocial behavior [41,42]. Secondly, social media can also serve as a crucial source of diverse social information that contributes to fostering “weak ties” among its users [43]. This characteristic effectively mitigates the proliferation of redundant information while simultaneously facilitating increased access to valuable insights into societal welfare initiatives (e.g., volunteer services). Thirdly, from a social network perspective, social media may enhance the users’ reservoir of social capital and expand their network of social relationships, thereby increasing the likelihood of accepting peer invitations to participate in specific prosocial activities [44]. Therefore, the following hypothesis is proposed:

**H3a:**  *Social media use has a positive impact on prosocial behavior in Chinese children and adolescents.*

Meanwhile, there is compelling evidence of a robust association between prosocial behavior and academic achievement [45]. Specifically, prior research has indicated that engaging in prosocial behavior could significantly enhance individual emotional and social cognitive abilities as well as psychological well-being [46,47]. In this case, students might better manage the stress associated with the learning process by bolstering their self-confidence and psychological resilience [14]. Furthermore, engaging in prosocial behavior also fosters a heightened sense of social connectedness [48]. This, in turn, could enhance students’ access to peer support and enable them to maintain a positive emotional state when faced with academic challenges. As such, it is likely that they would ultimately achieve improved academic performance [49]. Based on the aforementioned analysis and in conjunction with the inference of hypothesis H3a, it could be posited that the impact of social media use on academic performance may be mediated through prosocial behavior as an intermediary variable, thereby leading to the following hypotheses:

**H3b:**  *Prosocial behavior has a positive impact on academic performance in Chinese children and adolescents.*

**H3c:**  *Prosocial behavior plays a mediating role in the relationship between social media use and academic performance in Chinese children and adolescents.*

### 2.4. The Moderating Role of Family Socioeconomic Status

Childhood and adolescence are critical periods for the formation of personality, with socialization within the family environment having a long-term impact on individual development [50]. Given that parenting behavior is widely acknowledged as the primary means of implementing family socialization, disparities in family socioeconomic status may further contribute to variations in parenting practice. Drawing on Bourdieu’s theory of capitals, Lareau categorized parenting practice into two ideal types, i.e., “concerted cultivation” and “accomplishment of natural growth” [51]. More precisely, higher-class families tended to favor concerted cultivation, which emphasized achieving parental goals through active intergenerational communication and employing strict disciplinary measures in the process of child-rearing. On the contrary, lower-class families were more inclined toward the mode of natural growth, perceiving their children’s development as an organic progression and adopting a more lenient stance toward their children’s usage of social media. Considering the potential heterogeneity of parenting practice across different social classes, it is plausible that family socioeconomic status may exert an influence on the association between social media use and academic performance. Specifically, compared to families from a lower social class, those belonging to a higher social class tend to demonstrate significantly greater expectations and investments in their children’s education. As a result, parents with higher socioeconomic status are more likely to restrict their children’s non-study activities and allocate a substantial portion of their after-school time toward supplementary educational programs (e.g., shadow education). In other words, parents from higher social classes tend to assign more extracurricular tasks to their children, thereby limiting their engagement with social media activities. Therefore, in higher-class families, the elevated educational expectations of parents may, to some extent, weaken the effect of social media use on offsprings’ academic performance. Accordingly, we further propose the following hypothesis:

**H4:**  *Family socioeconomic status moderates the relationship between social media use and academic performance in Chinese children and adolescents.*

**Figure 1:**
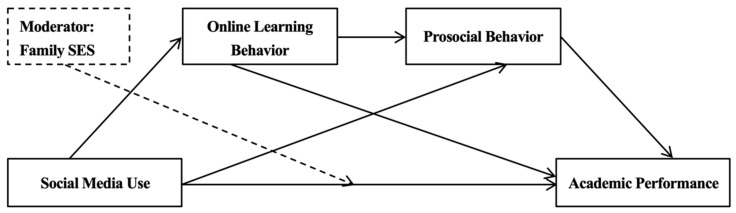
Theoretical model for this study.

It should be emphasized that online learning behavior and prosocial behavior are also interconnected and show a certain degree of positive association. Specifically, individuals’ online learning behavior can influence their knowledge accumulation and cognitive structure, direct their attention toward specific events or groups, and then enhance active participation in real-life prosocial behavior aimed at giving back to society [52]. Additionally, online learning platforms often encourage interpersonal communication and collaboration among users, which may cultivate an environment conducive to empathy development and promote their awareness of prosocial attitudes [53]. In a nutshell, based on the theoretical analyses, we constructed a moderated chain mediation model (see Figure 1).

## 3. Materials and Methods

### 3.1. Data Source

This study was based on the 2022 Chinese Minors’ Digital Life and Online Protection Survey, which was jointly organized by the Youth Rights Division of Central Committee of the Chinese Communist Youth League and the Institute of Sociology of the Chinese Academy of Social Sciences. An online questionnaire survey was conducted from May to July 2022 across 31 provinces in China, including autonomous regions and municipalities directly under the Central Government. The survey was designed to gather information regarding the digital literacy, psychosocial characteristics, and family education conditions of Chinese minors. Specifically, it employed a multi-stage stratified sampling method to investigate students aged between 6 and 18 years old, along with their parents. In the first stage, 3 to 4 prefecture-level cities were selected from each province. Then, four schools were chosen from each selected prefecture-level city, comprising two primary schools, one secondary school, and one high school/technical school. Within these selected schools, a random sampling approach was utilized to select one class per grade for conducting the questionnaire survey among the students in the selected class and their parents. After excluding respondents with missing data for key variables, a total of 8321 paired samples consisting of students and parents were ultimately retained for subsequent empirical analyses.

### 3.2. Measurement

The dependent variable of the current study was academic performance. We employed the following three items in the questionnaire to assess students’ academic performance: (1) What is your level of academic performance in class? (2) Are your parents satisfied with your academic performance? (3) Are you satisfied with your own academic performance? Among these, the first item gauged students’ objective academic achievement, while the latter two items measured it from both the parental and personal subjective perspectives. The possible responses to the three questions ranged from 1 to 5, with higher values indicating better academic performance. Principal component analysis was employed to extract a common factor from these three indicators, resulting in a cumulative variance contribution rate of 69.922%. Subsequently, for the sake of analytical convenience, we transformed the common factor into a continuous variable (ranging from 0 to 100) by means of the min–max standardization method.

Social media use served as the core explanatory variable in this study, encompassing a wide range of activities conducted through social media platforms. These activities included interactive communication, information sharing, leisure and entertainment, content consumption and production, and other behaviors exhibited by individuals or groups. Regarding the operationalization of social media use, the survey inquired about the frequency characteristics of the following relevant activities: visiting Sina microblogs, engaging with online communities (e.g., Baidu, Tieba, Zhihu), utilizing social networking sites, obtaining news and information online, expressing opinions on social events through digital platforms, discussing societal issues with others online, and supporting fan-based activities (e.g., voting for idols online). The items were rated on a five-point response scale ranging from “never = 1” to “always = 5”, yielding a Cronbach’s alpha coefficient of 0.860. We calculated the mean score across these seven items to assess the frequency of students’ usage of social media.

Regarding the operationalization of online learning behavior, the survey asked about the frequency at which the respondents engaged in the following activities: participation in online classes, completion of online homework assignments, utilization of online platforms for asking questions and seeking answers, retrieval of relevant online materials, memorization of English vocabulary through online means, engagement with online educational software, and acquisition of extracurricular knowledge through online sources. The respondents were asked to rate their responses on a five-point Likert scale ranging from “never = 1” to “always = 5”. The Cronbach’s alpha coefficient value of 0.884 was obtained through an analysis of internal consistency reliability. Then, we computed the mean score across these seven items to measure students’ online learning behavior.

Regarding the operationalization of prosocial behavior, we employed the following four items in the questionnaire for assessing students’ prosocial behavior over the past year: (1) monetary donations toward aiding individuals in distress; (2) active participation in volunteer service; (3) engagement in activities encompassing eco-friendly travel, water conservation, waste segregation, and reduction in plastic bag usage; and (4) assisting unfamiliar individuals. The response options for these items ranged from “never = 1” to “always = 7”, yielding a Cronbach’s alpha coefficient of 0.827. The scores of the four items were averaged to measure an individual’s level of prosocial behavior, with higher values indicating greater levels of prosociality.

In the statistical analyses, we incorporated the following control variables: the student’s gender (male = 1, female = 0); household registration (urban registration = 1, rural registration = 0); school attribute (key school = 1, non-key school = 0); stage of schooling (as proxy variables for age). Additionally, to control for the influence of family background, we further introduced the following two variables, i.e., parenting style and family socioeconomic status. Regarding parenting style, the questionnaire included a set of questions that assessed parents’ strictness in seven dimensions, including study arrangements, scholastic achievements, daily schedule, social interactions, attire choices, online engagement duration, and television consumption habits. The response options ranged from “no management = 1” to “very strict management = 5”. The Cronbach’s alpha coefficient for these items was calculated as 0.893. We averaged them to obtain the value of the presenting parenting style, with a higher value indicating a more stringent parenting approach. Regarding family socioeconomic status, we operationalized this variable by considering the educational levels of both fathers and mothers, as well as the annual income of the family. Principal component analysis was employed to extract a common factor from these three indicators, with a cumulative variance contribution rate of 65.206%. Then, we recoded the family socioeconomic status into a continuous variable (ranging from 0–100) using the min–max transformation method. The descriptive statistical results of the research variables are presented in Table 1.

### 3.3. Analytical Strategy

We employed SPSS26.0 and the PROCESS3.5 plug-in for statistical analysis, which was conducted in three steps. Firstly, descriptive methods were utilized to outline the fundamental distribution characteristics of Chinese students’ social media use and academic performance across different groups in the digital era. Secondly, based on a stepwise regression method and bootstrap mediation effect testing, we quantitatively analyzed the path relationships among social media use, online learning behavior, prosocial behavior, and academic performance in order to validate the aforementioned research hypotheses. Lastly, by incorporating the interaction term into the statistical model, we further examined the moderating effect of family socioeconomic status on the relationship between social media use and academic performance.

## 4. Research Results

### 4.1. Descriptive Statistics of Social Media Use and Academic Performance in Chinese Children and Adolescents

Currently, the overall level of social media use among Chinese children and adolescents remains relatively low, with an average frequency score of 1.646 (see Table 1). Table 2 presents, in detail, the distribution characteristics of social media use activities, revealing that over 70% of the surveyed students have never engaged in specific online activities, such as fan support (81.6%), discussing social issues online (71.8%), and expressing opinions on societal matters through online platforms (70.7%). In contrast to social media use, the mean score of online learning behavior was significantly higher at 2.685 (see Table 1), which suggests that Chinese parents tend to discourage non-educational internet usage due to concerns about diminishing their academic aspirations. Actually, parental close monitoring may, to a certain extent, impede students’ access to social media platforms and hinder the progress of digital education among children and adolescents.

Table 3 further demonstrates the status quo of students’ social media use and academic performance in different subgroups. The ANOVA results indicate that, compared to the reference group, female students with rural registration, enrolled in key schools, possessing a higher education level, and from a lower socioeconomic background exhibited a higher frequency of social media use. Meanwhile, those with urban registration, attending key schools, and from higher socioeconomic background showed relatively better academic performance. Moreover, Table 3 also reveals a less pronounced gender disparity and school-based variations in students’ academic performance, which reflects the ongoing trend toward gender equality and the equitable distribution of educational resources. Nevertheless, it is worth noting that both social media use and academic performance still appear to be associated with family socioeconomic status, thereby suggesting an inherent characteristic of social stratification within the field of minors’ development. In the subsequent section, we continue to carry out in-depth quantitative analyses to elucidate the underlying mechanism linking social media use and academic performance among Chinese children and adolescents.

### 4.2. Examination of Chain Mediation Effects

By utilizing the PROCESS plug-in of SPSS and employing the bootstrapping method proposed by Hayes [54], we conducted a stepwise regression analysis to empirically examine the research hypotheses proposed above. The results are presented in Table 4. First of all, after controlling for other variables, Model 1 demonstrated a statistically significant positive impact of social media use on academic performance (regression coefficient = 1.676, *p* < 0.01). Thus, H1 was validated. Then, Model 2 and Model 3, respectively, investigated the impacts of social media use on the two mediating variables. The findings indicate a significant positive relationship between social media use and online learning behavior (regression coefficient = 0.397, *p* < 0.01), as well as a similar positive association with prosocial behavior (regression coefficient = 0.264, *p* < 0.01), thereby providing support for hypotheses H2a and H3a. Finally, both the core independent variable and mediating variables were incorporated into Model 4. We found that there were significant positive effects of online learning behavior (regression coefficient = 0.988, *p* < 0.01) and prosocial behavior (regression coefficient = 1.723, *p* < 0.01) on academic performance, which further verified H2b and H3b.

Based on the results of the stepwise regression analyses presented in Table 4, we proceeded to employ the bootstrap method to conduct 5000 rounds of random sampling to examine the mediating effects of online learning behavior and prosocial behavior. The indirect effects of mediation paths and their proportion to the total effect are reported in Table 5. The statistical results show that the specific paths, i.e., SMU → OLB → AP and SMU → PB → AP, accounted for 23.4% and 27.1% of the total effect, respectively. Furthermore, SMU also exhibited a chained mediating effect upon academic performance through OLB and PB, contributing to 10.4% of the total effect. In terms of the relative effect sizes, the direct effect accounted for 39.1% of the total effect, while the combined effect of the three mediating effects explained 60.9% of the total effect, surpassing that of the direct effect. As indicated by Model 4 in Table 4, the direct effect of SMU → AP was statistically significant (regression coefficient = 0.655, *p* < 0.1), suggesting that OLB, PB, and OLB → PB partially mediated the relationship between social media use and academic performance. In other words, social media use could not only directly improve students’ academic performance but also exert substantial indirect effects through these two mediating factors, which validated H2c and H3c. Thus, the proposed chain mediation model for this study was supported by empirical materials.

### 4.3. Examination of the Moderating Effect of Family Socioeconomic Status

Previous literature points out that family socioeconomic status might exert heterogeneous influence on individuals’ socialization process [50,51]. Hence, does the identified promoting effect of social media use on students’ academic performance differ based on variations in their family background? Next, we introduced an interaction term based on the above mediation model, designed to further explore the moderating effect of family socioeconomic status. As shown in Model 5 of Table 6, the coefficient of the interaction term between family socioeconomic status and social media use was −0.032, with a bootstrap 95% confidence interval that did not include zero, indicating a statistically significant negative moderating effect. Therefore, H4 was supported.

**Figure 2:**
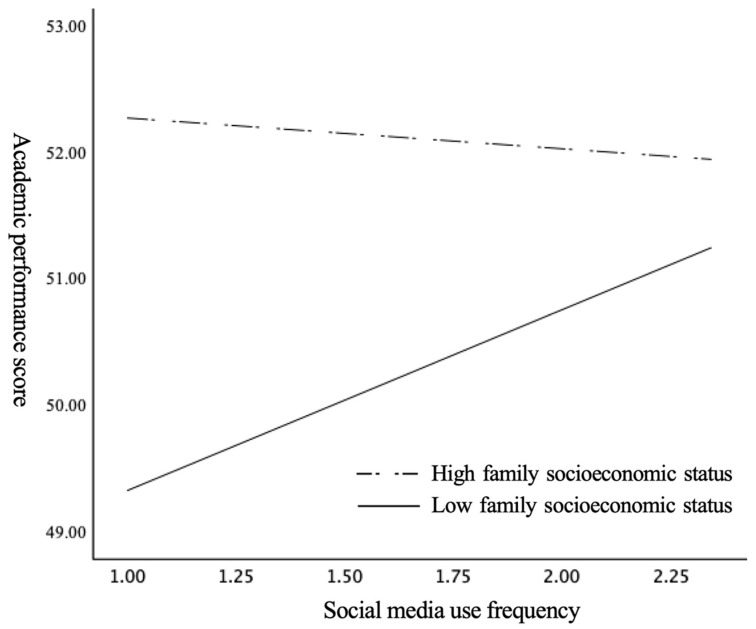
The simple slope diagram of family socioeconomic status.

**Figure 3:**
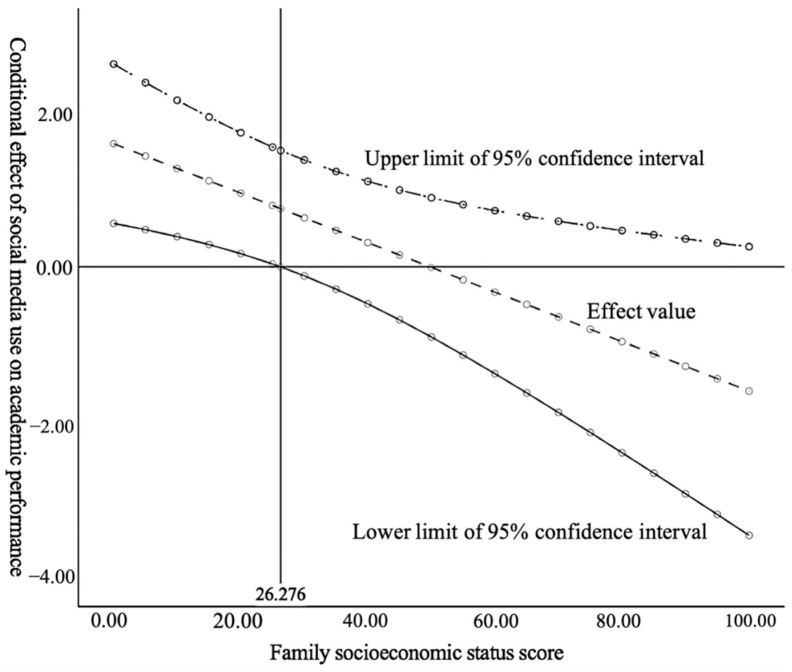
Johnson–Neyman conditional effect diagram of social media use.

To visually depict the moderating role of family socioeconomic status and its influencing boundary more effectively, we further drew a simple slope diagram and a Johnson–Neyman conditional effect diagram. As shown in Figure 2, compared to those with a high family socioeconomic status (i.e., mean + standard deviation), the impact of social media use on academic performance seemed to be significantly more pronounced among students with a low family socioeconomic status (i.e., mean − standard deviation). This finding suggests that students with limited family educational resources could compensate by leveraging individual capital acquired through social media, thereby enhancing their academic performance. In Figure 3, the Johnson–Neyman method was employed to quantitatively assess the threshold interval of the significant moderating effect of family socioeconomic status. The results indicate that when the family socioeconomic status index fell below 26.276 (i.e., cumulative percentile from low to high at about 50.8%), the confidence interval for the conditional effect remained positive but gradually diminished. However, when the family socioeconomic status index exceeded 26.276, the confidence interval for the conditional effect began to include zero, indicating that the moderating effect within this statistical range was no longer statistically significant. Thus, it can be inferred that the importance of utilizing social media as a means to enhance academic performance is decreased for those belonging to higher socioeconomic backgrounds. In this sense, if we consider academic performance as one of the determinants of individual holistic development, then students from lower socioeconomic families may leverage the relatively stronger marginal utility derived from social media usage, thereby aiding in mitigating developmental inequalities faced by underprivileged children due to familial disadvantages.

## 5. Conclusions, Implications, and Study Limitations

### 5.1. Conclusions

At present, the rapid proliferation and extensive application of digital technology have resulted in ubiquitous usage of social media platforms among school-aged children and adolescents worldwide. Admittedly, the openness of the cyber space evidently promotes educational fairness by providing children and adolescents with access to a wide range of learning resources, many of which are free. In light of this, the examination of social media’s impact on students’ academic development has emerged as a prominent topic in recent scholarly research. Drawing on data from the 2022 Chinese Minors’ Digital Life and Online Protection Survey, the current study empirically explored the status quo of social media use and its impact on academic performance among children and adolescents. By developing a moderated chain mediation model, we aimed to enrich our comprehension of the behavioral determinants influencing academic performance and contribute to addressing the issue of digital inequality in Chinese students’ educational advancement.

This study employed multiple regression analysis and the bootstrapping test method to analyze the effects of Chinese students’ social media use, online learning behavior, and prosocial behavior on their academic performance. The main research findings are as follows: Firstly, the average level of social media use among Chinese children and adolescents was generally low, while their academic performance exhibited certain heterogeneity in terms of socio-demographic and schooling characteristics. Secondly, after controlling for other factors, the frequency of social media use had a significant positive impact on students’ academic performance. Thirdly, the mechanism analysis revealed that online learning behavior and prosocial behavior served as significant mediators in the relationship between social media use and academic performance. For instance, students could transfer their social media behavioral patterns to the internet-based learning context, and then effectively utilize remote learning resources. Furthermore, engagement with social media could cultivate individuals’ prosocial personality, thereby stimulating intrinsic motivation for learning and ultimately enhancing academic performance. Fourthly, the heterogeneity analysis further confirmed that the impact of social media use on students’ academic performance was stronger in lower-class families, underscoring the significant moderating role of family socioeconomic status in the relationship between social media use and academic performance.

### 5.2. Theoretical and Practical Implications

From an academic standpoint, these findings make a theoretical contribution to the advancement of research in the domain of minors’ development. Specifically, by empirically validating the interplay among social media use, online learning behavior, and prosocial behavior, this study provides a novel analytical framework to comprehend the underlying mechanisms influencing students’ academic performance in the context of a digital society. In addition, the existing literature has highlighted disparities in the academic progress of children and adolescents stemming from their socioeconomic backgrounds [51], yet the potential functions arising from such disparities have remained inadequately explicated. By incorporating family socioeconomic status as a moderating variable, our study substantiates that the enhancing impact of social media use on academic performance tends to diminish with increases in the family socioeconomic status index. This finding reminds us that the rational utilization of social media could alleviate the familial disadvantages resulting from limited economic and cultural resources, thereby optimizing opportunities for upward social mobility among students hailing from lower socioeconomic backgrounds. Lastly, this study underlines the growing significance of digital media’s social construction function, particularly in light of the evolution of artificial intelligence and new media technologies. Indeed, the virtual socialization process based on cyberspace has become a parallel mechanism to real-life socialization processes. In this sense, this article also offers valuable insights for further exploration of socialization theory applicable to the digital age.

In view of the aforementioned empirical findings, we put forward the following policy recommendations. To begin with, it is crucial to actively enhance the policy support system aimed at providing high-quality online resources and fostering a conducive social environment for the rational utilization of social media by Chinese children and adolescents, considering their relatively limited engagement with such platforms. Policy makers should actively promote the establishment of relevant institutions to enhance the child-friendly features of social media. This will effectively curtail the dissemination of misinformation. Meanwhile, government departments should provide substantial support to digital literacy education programs, which will help enhance minors’ proficiency in utilizing social media as a means to acquire learning resources and foster knowledge-sharing habitus.

Next, given the promotional effect of social media use on academic performance, both parents and teachers, as crucial agents of early socialization, should actively explore the effective integration of social media into educational activities. For instance, platforms such as WeChat, Weibo, and TikTok can be effectively utilized to host online forums that facilitate the seamless sharing of educational resources between families and schools, exchange valuable educational experiences, and foster a consensus on education. These collaborative efforts have the potential to contribute significantly toward establishing a secure learning environment for minors. Additionally, school administrators ought to arrange regular training sessions to enhance the proficiency of teachers and parents in utilizing social media for educational purposes, encompassing the development and implementation of instructional activities based on social media as well as the management of students’ interactions within online learning environments [55,56,57].

Furthermore, this study emphasizes the importance of online learning behavior and prosocial behavior as influential predictors of students’ academic performance. As such, parents may deliberately devise personalized training plans for their children’s online learning, aid in optimizing time allocation, and facilitate their children’s participation in offline social activities through online platforms. They also need to actively participate in their children’s digital lives with a more responsible and inclusive attitude, thereby facilitating students’ better adaptation to the digital transformation of learning modes. To summarize, parents of varying socioeconomic backgrounds should acknowledge the benefits of networked social media on shaping a well-rounded personality and strive to explore a collaborative mechanism that integrates education and entertainment, which may help to effectively promote individuals’ comprehensive development.

### 5.3. Limitations and Prospects

There are still some limitations to this study. First, given that the statistical analyses in this article rely on cross-sectional survey data, it is challenging to establish causal relationships between the variables. Therefore, it is necessary to employ a longitudinal research design to reaffirm the robustness of the research findings. Second, the measurements of certain variables are not rigorous due to the limitation of questionnaire data, which may compromise the precision of research findings. Third, in the analysis of mechanisms, this study primarily focuses on elucidating the mediating role of individual behavioral factors, specifically online learning and prosocial behavior. However, it does not delve into exploring the intricate interplay among individuals, school environment, and peer groups, which may exert multifaceted influences on students’ academic performance. In future investigations, we will endeavor to overcome the above-mentioned limitations in order to attain a more precise comprehension of the heterogeneous mechanisms connecting social media use and academic performance among children and adolescents.

## Figures and Tables

**Table 1:** Descriptive statistics for the research variables.

Variables	Coding	Mean (Percentage)	Standard Deviation
Academic performance	0 to 100	51.253	20.817
Social media use	1 to 5	1.646	0.698
Online learning behavior	1 to 5	2.685	0.844
Prosocial behavior	1 to 7	2.992	1.290
Gender	Male = 1, female = 0	46.8%	-
Household registration	Urban registration = 1, rural registration = 0	48.8%	-
School attribute	Key school = 1, non-key school = 0	37.7%	-
Stage of schooling			
Primary education	Yes = 1, no = 0	41.0%	-
Secondary education	Yes = 1, no = 0	24.0%	-
High school/technical school education	Yes = 1, no = 0	35.0%	-
Parenting style	1 to 5	3.561	0.733
Family socioeconomic status	0 to 100	31.241	26.165

**Table 2:** The frequency distribution characteristics of students’ social media use activities.

	Never	Rarely	Sometimes	Often	Always
Visit Sina microblogs	67.5%	16.0%	11.5%	3.1%	1.9%
Engage with online communities	66.1%	16.7%	12.4%	3.0%	1.8%
Utilize social networking sites	49.1%	19.0%	19.1%	8.1%	4.7%
Obtain news and information online	35.5%	25.1%	28.1%	8.2%	3.1%
Support fan-based activities	81.6%	9.5%	6.6%	1.2%	1.1%
Express opinions on social events	70.7%	16.9%	9.7%	1.6%	1.1%
Discuss societal issues with others online	71.8%	15.9%	9.8%	1.5%	1.0%

**Table 3:** Subgroup distribution characteristics of social media use and academic performance.

Variables	Grouping	Social Media Use	Academic Performance	*n*
Gender	Male	1.576	51.445	3896
	Female	1.708	51.083	4425
	ANOVA test result	*p* < 0.01	*p* = 0.429	
Household registration	Urban	1.557	53.356	4063
	Rural	1.731	49.246	4258
	ANOVA test result	*p* < 0.01	*p* < 0.01	
School attended	Key School	1.681	50.693	3138
	Non-key School	1.625	51.592	5183
	ANOVA test result	*p* < 0.01	*p* = 0.056	
Education level	Primary education	1.283	55.901	3408
	Secondary education	1.716	48.980	1998
	High School/technical school education	2.023	47.377	2915
	ANOVA test result	*p* < 0.01	*p* < 0.01	
Family socioeconomic status	High family socioeconomic status	1.529	54.170	3630
	Low family socioeconomic status	1.737	48.995	4691
	ANOVA test result	*p* < 0.01	*p* < 0.01	

Notes: The mean values of social media use and academic performance are reported. Family socioeconomic status was categorized into a high/low group based on this variable’s mean value. The significance level of the F test for group comparisons was determined using the analysis of variance (ANOVA) method and reported accordingly.

**Table 4:** Stepwise regression analyses on the influencing paths of academic performance.

Variables	Model 1 (DV: Academic Performance)	Model 2 (DV: Online Learning Behavior)	Model 3 (DV: Prosocial Behavior)	Model 4 (DV: Academic Performance)
Constant	48.131 *** (1.507)	1.675 *** (0.059)	0.980 *** (0.096)	44.048 *** (1.578)
Gender (female = 0)	−0.096 (0.451)	−0.061 *** (0.018)	0.111 *** (0.027)	−0.199 (0.448)
Household registration (rural = 0)	1.062 ** (0.505)	0.014 (0.020)	−0.060 ** (0.031)	1.146 ** (0.502)
School attended (non-key school = 0)	−0.399 (0.470)	−0.018 (0.018)	0.097 *** (0.029)	−0.540 (0.467)
Education level (primary education = 0)				
Secondary education	−6.498 *** (0.624)	0.124 *** (0.024)	0.187 *** (0.038)	−6.998 *** (0.622)
High school/technical school education	−8.130 *** (0.642)	0.056 ** (0.025)	0.152 *** (0.039)	−8.472 *** (0.638)
Parenting attitude	0.911 *** (0.326)	0.094 *** (0.013)	0.189 *** (0.020)	0.452 (0.327)
Family socioeconomic status	0.039 *** (0.010)	0.001 (0.001)	0.002 *** (0.001)	0.035 *** (0.010)
Social media use	1.676 *** (0.364)	0.397 *** (0.014)	0.264 *** (0.023)	0.655 * (0.381)
Online learning behavior			0.257 *** (0.017)	0.988 *** (0.283)
Prosocial behavior				1.723 *** (0.179)
R^2^	0.042	0.116	0.080	0.056
F value	45.598 ***	136.016 ***	80.528 ***	48.833 ***

Notes: The standard error of the non-standardized regression coefficient is presented in parentheses; * denotes *p* < 0.1, ** denotes *p* < 0.05, and *** denotes *p* < 0.01. DV = dependent variable.

**Table 5:** Decomposition of indirect effects of social media use on academic performance.

Influence Paths	Indirect Effect Value	Bootstrap 95% CI	Percentage (Indirect Effect/Total Effect)
SMU → OLB → AP	0.392	[0.155, 0.631]	23.4%
SMU → PB → AP	0.454	[0.328, 0.598]	27.1%
SMU → OLB → PB → AP	0.175	[0.129, 0.227]	10.4%

Notes: The total effect size was 1.676, with a bootstrap 95% confidence interval ranging from 0.962 to 2.391. SMU = social media use, OLB = online learning behavior, PB = prosocial behavior, AP = academic performance.

**Table 6:** Test results of the moderating effect of family socioeconomic status.

DV: Academic Performance	Model 5	Bootstrap 95% CI
Social media use	1.591 (0.525)	[0.561, 2.620]
Online learning behavior	1.027 (0.284)	[0.471, 1.583]
Prosocial behavior	1.726 (0.179)	[1.375, 2.077]
Family socioeconomic status	0.088 (0.023)	[0.044, 0.133]
Family socioeconomic status × social media use	−0.032 (0.012)	[−0.056, −0.008]
Other variables	Yes	

Notes: The settings of other variables in this table remained consistent with those presented in Table 4. DV = dependent variable; CI = confidence interval.

## Data Availability

The datasets generated for this study will not be made publicly available as the authors do not have permission to share the data.
